# Identifying sequence variants contributing to hereditary breast and ovarian cancer in *BRCA1* and *BRCA2* negative breast and ovarian cancer patients

**DOI:** 10.1038/s41598-019-55515-x

**Published:** 2019-12-27

**Authors:** Elisabeth Jarhelle, Hilde Monica Frostad Riise Stensland, Geir Åsmund Myge Hansen, Siri Skarsfjord, Christoffer Jonsrud, Monica Ingebrigtsen, Nina Strømsvik, Marijke Van Ghelue

**Affiliations:** 1 Department of Medical Genetics, Division of Child and Adolescent Health, University Hospital of North Norway, Tromsø, Norway; 2 Department of Clinical Medicine, University of Tromsø, Tromsø, Norway; 3 Northern Norway Family Cancer Center, Department of Medical Genetics, University Hospital of North Norway, Tromsø, Norway; 4 Department of Health and Caring Sciences, Western Norway University of Applied Sciences, Bergen, Norway

**Keywords:** Breast cancer, Cancer genetics, Genetics research

## Abstract

Families with breast and ovarian cancer are often tested for disease associated sequence variants in *BRCA1* and *BRCA2*. Pathogenic sequence variants (PVs) in these two genes are known to increase breast and ovarian cancer risks in females. However, in most families no PVs are detected in these two genes. Currently, several studies have identified other genes involved in hereditary breast and ovarian cancer (HBOC). To identify genetic risk factors for breast and ovarian cancer in a Norwegian HBOC cohort, 101 breast and/or ovarian cancer patients negative for PVs and variants of unknown clinical significance (VUS) in *BRCA1/2* were screened for PVs in 94 genes using next-generation sequencing. Sixteen genes were closely scrutinized. Nine different deleterious germline PVs/likely pathogenic variants (LPVs) were identified in seven genes in 12 patients: three in *ATM*, and one in *CHEK2, ERCC5*, *FANCM*, *RAD51C*, *TP53* and *WRN*. Additionally, 32 different VUSs were identified and these require further characterization. For carriers of PV/LPV in many of these genes, there are no national clinical management programs in Norway. The diversity of genetic risk factors possibly involved in cancer development show the necessity for more knowledge to improve the clinical follow-up of this genetically diverse patient group.

## Introduction

A total of 3,589 new female breast cancer (BC) cases and 520 new ovarian cancer (OC) cases were reported in Norway in 2017^1^, 5–10% are thought to be due to inherited pathogenic variants (PVs)^2^. Since 1994 when *BRCA1* and *BRCA2* were identified^3,4^, PVs in these two genes have been known to be the leading cause of hereditary breast and ovarian cancer (HBOC). Together, mutated *BRCA1* and *BRCA2* are responsible for about 15–25% of familial breast and ovarian cancer cases^5,6^. The risk estimates for PVs in these genes are 45–65% for BC and 11–44% for OC by age 70^7^. Currently, approximately 3,000 *BRCA1* variants and 3,400 *BRCA2* variants are listed in ClinVar as PVs or likely pathogenic variants (LPVs) (https://www.ncbi.nlm.nih.gov/clinvar ^8^). Nevertheless, in a large proportion of HBOC families no PVs/LPVs in *BRCA1/2* have been identified.

Next-generation sequencing (NGS) allows for rapid screening of several genes and with this technology several variants in other genes have been linked to increased risk of BC and/or OC. The largest study of its kind, so far, investigated 35,409 women with a single breast cancer diagnosis, where 93.2% met the National Comprehensive Cancer Network (NCCN) guidelines for HBOC genetic testing^9^. These patients were screened for PVs using a 25-gene panel, and identified PVs/LPVs in 9.3%. Nearly half (48.5%) of the identified PVs/LPVs were located in *BRCA1* and *BRCA2*, meaning that more than half (51.5%) of all pathogenic findings were in other genes. Among the genes most frequently identified with PVs/LPVs were *CHEK2*, *ATM* and *PALB2*^9^. Additional studies have identified these three genes as the most frequently mutated after *BRCA1/2*^10,11^. In general, genes encoding proteins involved in homologous recombination repair, the same pathway in which BRCA1 and BRCA2 are involved, are frequently reported with pathogenic findings in HBOC cases. These genes include the previously mentioned *CHEK2, ATM* and *PALB2*, together with *NBN, RAD50, RAD51C, RAD51D* and *BRIP1*^9,10,12–14^. In addition, PVs in genes from the overlapping Fanconi Anaemia (FA) pathway and mismatch repair (MMR) pathway have been identified in BC and OC patients^7^. Several NGS studies revealing PVs/LPVs in other genes than *BRCA1/2* in HBOC cancer patients have been published over the last years^9–19^. However, no such study has been reported on HBOC patients in Norway. Identification of the population-specific mutation spectrum is critical, since accumulation of certain genetic aberrations may occur within a population. In the present study, we included Norwegian women diagnosed with BC and/or OC, for whom no *BRCA1* or *BRCA2* PV/LPV/variant of unknown clinical significance (VUS) have been identified.

## Results

**Figure 1:**
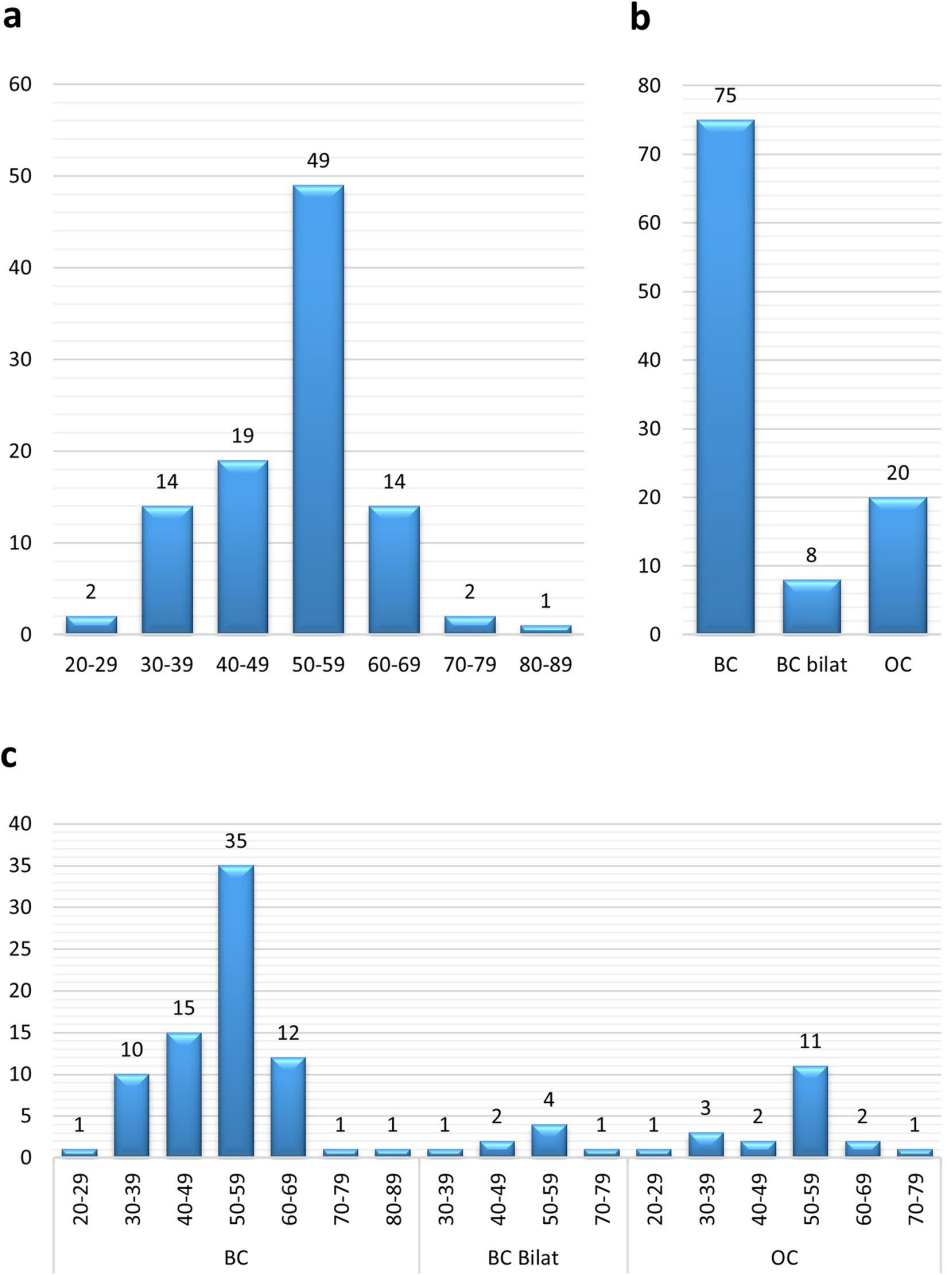
Distribution of patient age and diagnosis. (**a**) Age distribution upon first BC/OC diagnosis, regardless of diagnosis. (**b**) Patients grouped according to BC/OC diagnosis. One patient presented both with BC and OC (P-18), another patient (P-68) had both bilateral BC and OC. Accordingly, these two patients were registered in both BC and OC or BC bilat and OC patient groups, respectively. (**c**) Combination of age and BC/OC diagnosis of patients. P-18 and P-68 are also here represented twice. BC = breast cancer. Bilat = bilateral. OC = ovarian cancer.

A total of 101 BC and/or OC patients with no *BRCA1/2* PVs, LPVs or VUSs were included in the study (Supplementary Table S1). In Fig. 1, diagnosis and age at onset represented in 10-year intervals are displayed. The majority of the patients were diagnosed at age 50–59 in all three diagnosis groups (BC, bilateral BC and OC).

Samples from the 101 patients were investigated for the presence of nonsense or frameshift variants in 94 genes. In addition, 16 genes were scrutinized for missense, deletions, insertions, and possible splice-affecting variants (Table 1). The average coverage of the examined regions for samples from group 1 and 2 was 532.7 reads (S.D. 138.9). The average coverage information was not available for samples in group 3 where only Virtual Contact File (VCF) and Binary Alignment Map (BAM) files were studied. However, since all three sample groups were analyzed in an identical manner, similar coverage can be inferred.

**Table 1:** Overview of the 18 genes (including *BRCA1* and *BRCA2*) investigated more closely in this study (the 18 first genes in the table) together with four genes with additional findings^a^.

Gene symbol	Gene name	Reference sequence	Numbers of exons	Average coverage	Gaps
*BRCA1*	BRCA1, DNA repair associated	NM_007294.3	23	514.6	
*BRCA2*	BRCA2, DNA repair associated	NM_000059.3	27	737.7	
*ATM*	ATM serine/threonine kinase	NM_000051.3	63	795.6	
*BRIP1*	BRCA1 interacting protein C-terminal helicase 1	NM_032043.2	20	714.6	
*CDH1*	Cadherin 1	NM_004360.3	16	527.9	Ex2/in2: 63% 5′ in11: 16%
*CHEK2*	Checkpoint kinase 2	NM_007194	16	592.7	
*MLH1*	MutL homolog 1	NM_000249.3	19	573.1	
*MSH2*	MutS homolog 2	NM_000251.2	16	736.1	
*MSH6*	MutS homolog 6	NM_000179.2	10	697.8	Ex1/in1: 100%
*NBN*	Nibrin	NM_002485.4	16	764.3	
*NF1*	Neurofibromin 1	NM_001042492.2	58	594.8	
*PALB2*	Partner and localizer of BRCA2	NM_024675.3	13	578.8	
*PMS2*	PMS1 homolog 2, mismatch repair system component	NM_000535.6	15	735.3	
*PTEN*	Phosphatase and tensin homolog	NM_00314.4	9	643.6	
*RAD51C*	RAD51 paralog C	NM_058216.1	9	641.8	
*RAD51D*	RAD51 paralog D	NM_002878.3	10	447.0	In4/ex5: 94% Start of ex1: 89%
*STK11*	Serine/threonine kinase 11	NM_000455.4	10	317.9	In3/ex4: 13% Ex7/in7: 54% Ex9/in9: 32%
*TP53*	Tumor protein 53	NM_000546.5	11	414.2	3′ in6: 5%
*ERCC5*^a^	ERCC excision repair 5, endonuclease	NM_000123.3	15	683.6	
*FANCF*^a^	Fanconi anemia, complementation group F	NM_022725.3	1	744.0	
*FANCM*^a^	Fanconi anemia, complementation group M	NM_020937.2	23	866.8	
*WRN*^a^	Werner syndrome RecQ like helicase	NM_000553.4	35	679.8	

Reported gaps (<30 reads) are listed in the last column, the percentage in the Gaps column represents the amount of samples with gaps in this region (gaps only present in one run or in other genes than the genes more closely scrutinized are not included). In = intron. Ex = exon.

^a^Genes only investigated for frame-shift and nonsense variants and with an identified pathogenic/likely pathogenic variant in this study.

### Identified variants

**Figure 2:**
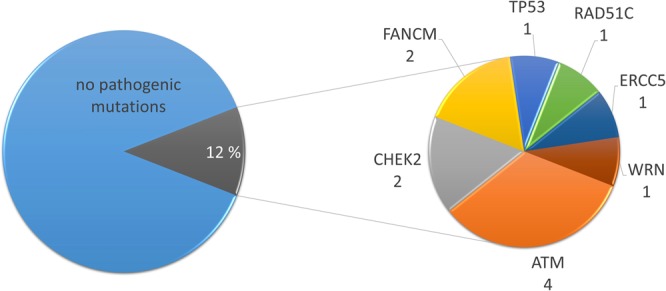
Distribution of patients with and without pathogenic/likely pathogenic variants. Twelve out of 101 patients were identified with pathogenic/likely pathogenic variants.

For the analysed regions, on average 203.4 variants (range: 170–247) in all 94 genes were reported per patient. After filtration, on average 1.1 variant (range: 0–4) per patient remained, which resulted in a total of 77 unique variants. Of these 77 variants, nine were classified as PVs/LPVs (Table 2), 32 classified as VUSs (Table 3) and 36 were classified as benign/likely benign (Supplementary Table S2). The nine unique PVs/LPVs were found in seven genes (*ATM, CHEK2, ERCC5, FANCM, TP53, RAD51C* and *WRN)* in 12 patients (Table 2 and Fig. 2).

**Table 2:** Pathogenic and likely pathogenic variants identified in a Norwegian breast and ovarian cancer cohort.

Gene	Variant	Local.	Protein change	Databases				Pr. Ref.	Cl.	Patient	Diagn. & age	Additional VUS
				dbSNP	gnomAD	ClinVar	HGMDp					
*ATM*	c.3245_3247delinsTGAT	exon 22	p.His1082Leufs*14	—	—	RCV000159638.7; Pathogenic	CX983261; A-T: DM	^66^	5	P-31	OC, 47y	*BRIP1* c.2087C>T
*ATM*	c.3245_3247delinsTGAT	exon 22	p.His1082Leufs*14	—	—	RCV000159638.7; Pathogenic	CX983261; A-T: DM	^66^	5	P-2	BC, 57y	*NF1* c.5225A>G
*ATM*	c.5932G>T	exon 40	p.Ser1974Ilefs*4	rs587779852	ALL: 0.00%; NFE: 0.01%; FIN: 0%	RCV000115219.8; Pathogenic	CM980147; A-T: DM	^37^	5	P-91	BC, 54y	*NF1* c.469A>G
*ATM*	c.8432delA	exon 58	p.(Lys2811Serfs*46)	rs759472682	ALL: 0.00%; NFE: 0.00%; FIN: 0%	RCV000131776.5; Pathogenic	CD000916; A-T: DM	^67^	5	P-62	OC, 38y	—
*CHEK2*	c.319+2T>A	intron 2	p.(?)	rs587782401	ALL: 0.01%; NFE: 0.01%; FIN: 0.06%	RCV000131434.11; Likely pathogenic	CS1617635; BC: DM	^16^	4	P-12	OC, 27y	*CDH1* c.136C>G
*CHEK2*	c.319+2T>A	intron 2	p.(?)	rs587782401	ALL: 0.01%; NFE: 0.01%; FIN: 0.06%	RCV000131434.11; Likely pathogenic	CS1617635; BC: DM	^16^	4	P-16	OC, 70y	—
*ERCC5*	c.67G>T	exon 1	p.(Glu23*)	—	—	—	—	—	4	P-44	BC, 49y	*NF1 c.378A>G*
*FANCM*	c.5101C>T	exon 20	p.(Gln1701*)	rs147021911	ALL: 0.13%; NFE: 0.11%; FIN: 0,81%	RCV000115190.8; Risk factor^b^	CM147953; TF: DM	^68^	4	P-8	BC, 56y	—
*FANCM*	c.5101C>T	exon 20	p.(Gln1701*)	rs147021911	ALL: 0.13%; NFE: 0.11%; FIN: 0,81%	RCV000115190.8; Risk factor^b^	CM147953; TF: DM	^68^	4	P-41	BC, 69y	—
*RAD51C*	c.1026+5_1026+7delGTA	intron 8	p.Arg322Serfs*22	rs747311993	ALL: 0.00%; NFE: 0.00%; FIN: 0%	RCV000116170.11; Likely pathogenic	CD1313340; BOC: DM	^69^	4	P-69	OC, 52y	—
*TP53*	c.818G>A^a^	exon 8	p.(Arg273His)	rs28934576	ALL: 0.00%; NFE: 0.00%; FIN: 0%	RCV000115738.8; Pathogenic/Likely pathogenic	CM920677; LFS: DM	^70^	5	P-13	BC, 36y	—
*WRN*	c.1105C>T	exon 9	p.(Arg369*)	rs17847577	ALL: 0.02%; NFE: 0.03%; FIN: 0.02%	RCV000005782.8; Pathogenic^c^	CM961463; WRN: DM	^71^	5	P-90	BC, 57y	—

Variants are named according to Human Genome Variation Society (HGVS) nomenclature. Local.: localization; Pr.Ref: Primary reference; cl.: class (4: likely pathogenic; 5: pathogenic); diagn.: diagnosis; NFE: Non-Finnish Europeans; FIN: Finnish Europeans; A-T: Ataxia telangiectasia; DM: Disease mutation; BC: Breast cancer; OC: Ovarian cancer; LFS: Li-Fraumeni Syndrome; DFP: Disease associated functional polymorphism; TF: Tetralogy of Fallot; WRN: Werner syndrome; y: years. The ClinVar references and the corresponding clinical significance are mainly linked to the condition “Hereditary cancer-predisposing syndrome”, exceptions are marked.

^a^Variant identified in 33% of reads.

^b^Fanconi anemia.

^c^Werner syndrome.

**Table 3:** Variants of unknown clinical significance identified in a Norwegian breast and ovarian cancer cohort.

Gene	Variant	Local.	Protein change	Databases				Pr. Ref.	Patient	Diagn. & age
				dbSNP	gnomAD	ClinVar	HGMDp			
ATM	c.1727T>C	Exon 11	p.(Ile576Thr)	rs730881342	ALL: 0.01%; NFE: 0.01%; FIN: 0.04%	RCV000159685.6; VUS	—	^72^	P-97	BC, 50y
ATM	c.1986T>C	Exon 13	p.(=)	rs1800055	ALL: 0.05%; NFE: 0.08%; FIN: 0.08%	RCV000123724.5; Likely benign	—	^72^	P-99	BC, 52y
ATM	c.2164T>C	Exon 14	p.(=)	—	—	—	—	—	P-78	BC, 49y
ATM	c.2220A>C	Exon 14	p.(=)	—	—	—	—	—	P-56	BC, 61y
ATM	c.2804C>T	Exon 19	p.(Thr935Met)	rs3218708	ALL: 0.01%; NFE: 0.01%; FIN: 0%	RCV000131651.8; Likely benign/VUS	CM177861; CRC: DM?	^73^	P-53	BC, 38y
ATM	c.3549T>C	Exon 24	p.(=)	rs767377764	ALL: 0.00%, NFE: 0%; FIN: 0%	RCV000223274.2; Likely benign	—	—	P-88	BC, 48y
ATM	c.3703C>T	Exon 25	p.(Pro1235Ser)	rs779095853	ALL: 0.00%, NFE: 0.00%; FIN: 0%	RCV000567940.1; VUS	—	—	P-80	BC, 58y
ATM	c.4324T>C	Exon 30	p.(Tyr1442His)	rs201666889	ALL: 0.03%; NFE: 0.06%; FIN: 0.02%	RCV000115190.8; Benign/VUS	CM0910502; BC: DM?	^30^	P-75	BC, 49y
ATM	c.5229A>G	Exon 36	p.(=)	—	—	RCV000233826.2; Likely benign^a^	—	—	P-42	BC, 63y
ATM	c.8734A>G	Exon 61	p.(Arg2912Gly)	rs376676328	ALL: 0.02%; NFE: 0.04%; FIN: 0.00%	RCV000131723.10; VUS	CM014034; BC: DM	^74^	P-14	BC, 35y
BRIP1	c.2087C>T	Exon 14	p.(Pro696Leu)	rs147755155	ALL: 0.00%; NFE: 0.01%; FIN: 0.00%	RCV000116135.9; VUS	—	—	P-31; P-32	OC, 47y; OC, 52y
CDH1	c.136C>G	Exon 2	p.(Leu46Val)	—	—	—	—	—	P-12	OC, 27y
CHEK2	c.470T>C	exon 4	p.(Ile157Thr)	rs17879961	ALL: 0.49%; NFE: 0.53%; FIN: 2.50%	RCV000116018.12; Likely pathogenic /Pathogenic	CM993368; LFS, IR: DFP	^75^	P-59	BC, 58y
CHEK2	c.538C>T	Exon 4	p.(Arg180Cys)	rs77130927	ALL: 0.09%; NFE: 0.12%; FIN: 0.03%	RCV000116024.8; Benign/VUS	CM030417; PC: DM	^76^	P-66	BC, 53y
CHEK2	c.1205_1206delinsTC	Exon 11	p.(Ala402Val)	—	—	RCV000537997.1; VUS^e^	—	—	P-74	BC, 54y
FANCF	c.1087C>T	Exon 1	p.(Gln363*)	rs201285915	ALL: 0.01%; NFE: 0.01%; FIN: 0%	RCV000482395.1; VUS^b^	CM1824108; BOC: DM	^20^	P-48	BC, 39y
MLH1	c.1665T>C	Exon 14	p.(=)	rs749204990	ALL: 0.00%, NFE: 0.00%; FIN: 0%	RCV000167487.2; Likely benign	—	—	P-65	BC bilateral, 51y
MSH2	c.1667T>C	Exon 11	p.(Leu556Ser)	rs587779101	—	RCV000076234.2; VUS^c^	CM148293; LS: DM?	^77^	P-73	BC, 50y
MSH2	c.2503A>C	Exon 15	p.(Asn835His)	rs41295296	ALL: 0.00%; NFE: 0.01%; FIN: 0%	RCV000115519.10; Likely benign; VUS	—	^78^	P-82	BC, 37
MSH6	c.3259C>T	Exon 5	p.(Pro1087Ser)	rs63750998	ALL: 0.01%; NFE: 0.02%; FIN: 0%	RCV000074827.4; VUS^c^	CM1210418; OC: DM	^79^	P-81	BC, 64y
NBN	c.643C>T	Exon 6	p.Arg215Trp	rs34767364	ALL: 0.25%; NFE: 0.40%; FIN: 0.32%	RCV000115802.12; Benign/Likely benign/ VUS	CM044022; CRC: DM	^80^	P-75	BC, 49y
NF1	c.378A>G	Exon 4	p.(=)	—	—	——	—	—	P-44	BC, 49y
NF1	c.469A>G	Exon 4	p.(Ile157Val)	—	—	RCV000566319.1; VUS	—	—	P-91	BC, 54y
NF1	c.587-6_587-5delTT	Intron 5	p.(?)	—	—	—	—	—	P-15	BC bilateral, 74/80y
NF1	c.960T>A	Exon 9	p.(=)	rs376447070	ALL: 0.02%; NFE: 0.01%; FIN: 0.17%	RCV000167230.1; likely benign	—	—	P-64	BC, 35y
NF1	c.4926A>G	Exon 37	p.(=)	—	—	—	—	—	P-88	BC, 48y
NF1	c.5225A>G	Exon 37	p.(Asn1742Ser)	rs745407845	ALL: 0.00; NFE: 0.00%; FIN: 0%	RCV000206576.4; VUS^d^	CM1512958; NF1: DM	^81^	P-2	BC, 57y
NF1	c.5793T>C	Exon 39	p.(=)	rs779114598	ALL: 0.00%; NFE: 0.01%; FIN: 0%	RCV000167490.1; likely benign	—	^82^	P-22	BC, 86y
NF1	c.7354C>T	Exon 50	p.(Arg2452Cys)	rs377662483	ALL: 0.00; NFE: 0%; FIN: 0%	RCV000203720.5; VUS^d^	—	—	P-1	BC, 36
NF1	c.7595C>T	Exon 51	p.(Ala2532Val)	rs148154172	ALL: 0.07%; NFE: 0.05%; FIN: 0.00%	RCV000130730.3; Likely benign	—	—	p-89	BC, 47y
PMS2	c.1765G>C	Exon 11	p.(Asp589His)	rs749727182	ALL: 0.00%; NFE: 0.00%; FIN: 0%	RCV000483031.2; VUS^b^	—	—	P-49	BC, 41y
PMS2	c.1936A>C	Exon 11	p.(=)	rs369582237	ALL: 0.00%; NFE: 0.01%; FIN: 0%	RCV000163542.2; likely benign	—	—	P-67	BC, 51y

Variants are named according to Human Genome Variation Society (HGVS) nomenclature. Local.: localization; Pr.Ref: Primary reference; diagn.: diagnosis; NFE: Non-Finnish Europeans; FIN: Finnish Europeans; DM: Disease mutation; CRC: colorectal cancer BC: Breast cancer; BOC: Breast and ovarian cancer syndrome; PC: Prostate cancer; CRC: Colorectal cancer; LS: Lynch syndrome; OC: Ovarian cancer; NF1: Neurofibromatosis, type 1; LFS,IR: Li-Fraumeni Syndrome, increased risk; DFP: Disease associated functional polymorphism; y: years. P-75 has two VUS’s, one in *ATM* and one in *NBN*. The two *MLH1* variants are listed together since they only have been identified in cis in Norway. The ClinVar references and the corresponding clinical significance are mainly linked to the condition “Hereditary cancer-predisposing syndrome”, exceptions are marked.

^a^Ataxia telangiectasia.

^b^AllHighlyPenetrant.

^c^Lynch syndrome reviewed by expert panel (InSiGHT).

^d^Neurofibromatosis, type 1.

^e^Familieal cancer of breast.

### Variants according to diagnosis and age-groups

Four of the 12 patients with PVs/LPVs findings were diagnosed with BC between ages 50–59 years. Additional three patients with PVs/LPVs were diagnosed with BC between 30–39 years, 40–49 years and 60–69 years old. Five PVs/LPVs were identified in patients diagnosed with ovarian cancer. Interestingly, no PVs/LPVs/VUSs were identified in two patients diagnosed with both OC and BC (P-18 and P-68). In P-68 the only identified variant passed filtration was the likely benign *MLH1* c.-7C>T, in *cis* (confirmed by manual investigation in the Integrative Genomics Viewer (IGV)) with *MLH1* c.-28A>G (c.[-28A>G; -7C>T]) (Supplementary Table S2) and none were identified in P-18.

### Variants in genes exclusively investigated for frameshift and stop variants

Five different variants were detected in the genes that were exclusively investigated for frameshift and stop variants. These five variants were located in *ERCC5, FANCF, FANCM* and *WRN* and were found in P-8, P-41, P-44, P-48 and P-90 (Tables 2 and 3). One of the patients (P-48) had two nonsense variants, in two different genes, *FANCF* c.1087C>T p.(Gln363*) and *WRN* c.4216C>T p.(Arg1406*). The *FANCF* variant was classified as VUS (Table 3) as this gene consists of only one exon and the variant therefor presumably results in the loss of the terminal 12 amino acids of a protein region, instead of undergoing nonsense mediated mRNA decay (NMD). However, the variant has a low population allele frequency in gnomAD (0.0071%) and has previously been reported in the literature as pathogenic by Quezada Urban *et al*.^20^. In ClinVar, it is reported as a VUS. The *WRN* c.4216C>T was classified as non-pathogenic due to its high allele frequency in the South Asian population (1.7% and 10 homozygotes in gnomAD). Additionally, this variant was classified as benign in ClinVar and listed as “DM?” (“Disease causing mutation?” = Variant reported as likely disease causing, but with questionable pathogenicity) in the Human Gene Mutation Database (HGMDp).

### Two interesting cases

**Figure 3:**
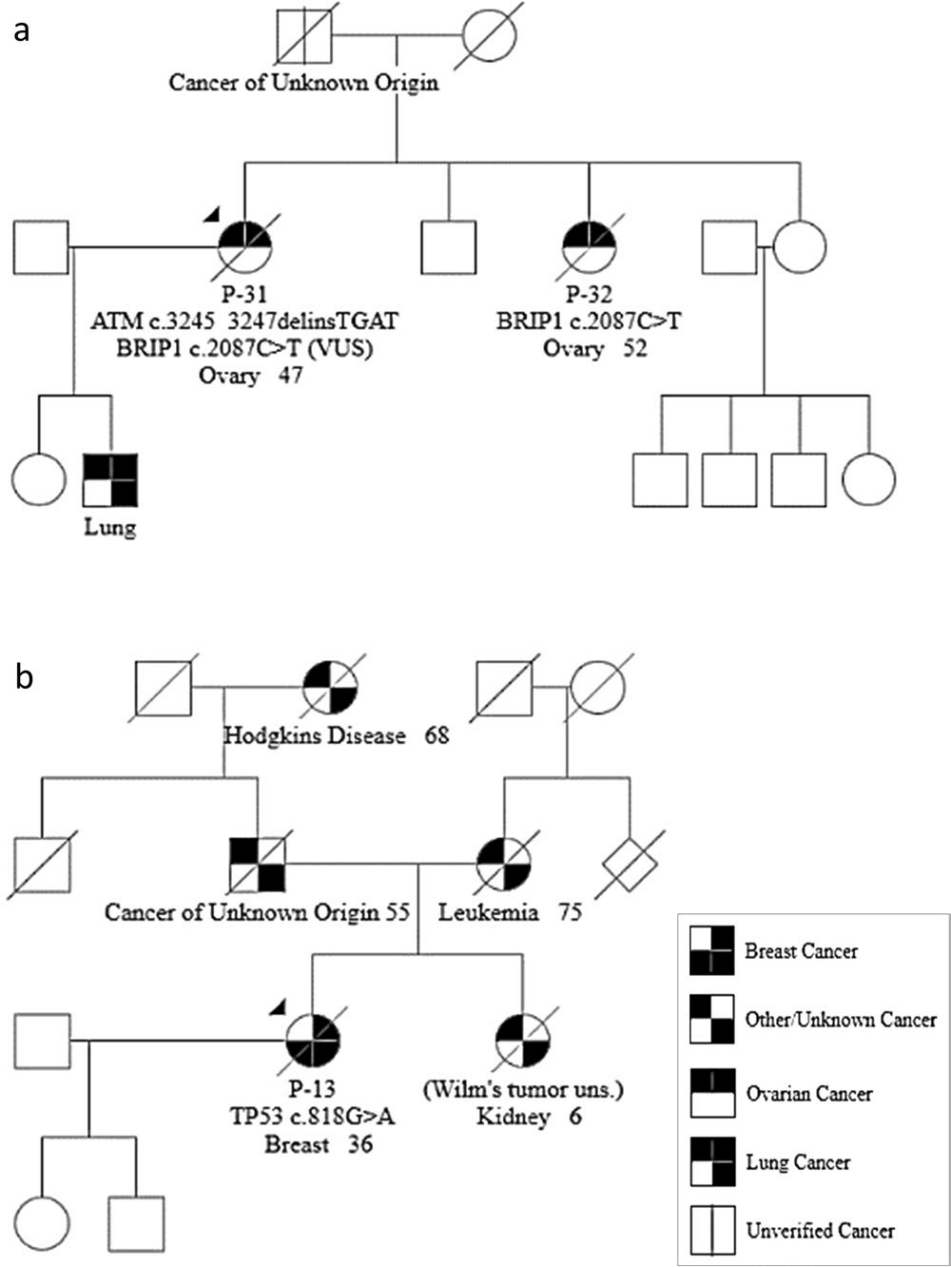
Illustration of two pedigrees. (**a**) Pedigree of P-31 and P-32 (*ATM* c.3245_3247delinsTGAT and *BRIP1* c.2087C>T). (**b**) Pedigree of P-13 (*TP53* c.818G>A).

Five of the 12 patients with a PV/LPV also had a VUS in one of the 16 genes more closely scrutinized (Tables 1 and 2). One of these patients (P-31) was diagnosed with OC and was heterozygous for the pathogenic sequence variant c.3245_3247delinsTGAT in *ATM*. Her sister (P-32), who was equally diagnosed with OC and likewise included in this study, did not have this variant. However, both sisters were heterozygous for a VUS in *BRIP1* (c.2087C>T p.(Pro696Leu)). Figure 3 depicts the pedigree of the two sisters (P-31 and P-32).

The *TP53* variant identified in this study was the pathogenic c.818G>A. This variant was identified in patient P-13, whose pedigree does not resemble a classic Li-Fraumeni family (Fig. 3).

## Discussion

In this study, 101 patients were screened for the presence of deleterious sequence variants in 94 cancer associated genes. We identified PVs/LPVs in 12 patients, in seven different genes (Table 2 and Fig. 2). In total, 9 different PV/LPVs were identified, including one novel sequence variant in *ERCC5* (c.67G>T). In addition, we detected 32 VUSs including six variants not previously described (Table 3).

These findings correspond to a total finding percentage of 12% in the investigated patient cohort (Fig. 2), which was in concordance with Pinto *et al*., Aloraifi *et al*. and Schubert *et al*.^11,15,21^. However, these finding percentages were higher than for several other studies (ranging from 4.7–9% in *BRCA1/2*-negative patients)^9,14,16–19,22–24^. In several of these studies, the majority of PVs/LPVs were identified in *ATM, CHEK2* and *PALB2*. In our study, the majority of the PVs/LPVs were identified in *ATM* and *CHEK2*, in four and two patients, respectively (Fig. 2). However, whereas several studies identified PVs in *PALB2*, we did not detect any. Comparison of these studies was challenging, since the gene panels and/or the studied cohort differed between most of them.

The study included in total 83 patients with BC and 20 patients with OC (two overlapping, diagnosed with both BC and OC) (Fig. 1). Seven of the patients diagnosed with BC were found to carry a PV/LPV, corresponding to findings in 8.4%. Furthermore, five of the 20 patients (25%) diagnosed with OC were found to carry a PV/LPV. These percentages correspond well with the estimated disease burden of BC and OC cases due to inherited PV/LPVs, 5–10% and ~25%, respectively^25,26^. However, in the current study, a small number of patients were included and caution should be taken when comparing with other studies. Screening of a larger amount of patients might change the observed finding percentage.

One of the most frequently identified mutated genes in the studied cohort was *ATM*. Biallelic deleterious *ATM* variants cause Ataxia Telangiectasia (A-T)^27^. However, heterozygous carriers of deleterious variants have an increased risk of breast cancer^28–32^. Although we do not diagnostically test for *ATM* variants in HBOC families, patients with a family history of BC/OC and an identified PV/LPV in *ATM* (sequenced elsewhere) are offered additional follow-up, including mammography from 40 years of age. This has been established in concordance with the other Departments of Medical Genetics in Norway. However, the cancer risk of PVs in *ATM* may still be debatable. Patient P-2, diagnosed with BC at 57 years of age, was a carrier of the pathogenic *ATM* c.3245_3247delinsTGAT (Table 2). This variant has previously been identified as a pathogenic variant and is a Norwegian founder mutation^33^. The same variant was also identified in a patient diagnosed with OC (P-31). The latter patient had a sister (P-32) who was diagnosed with OC at age 52. However, the *ATM* c.3245_3247delinsTGAT variant was exclusively present in P-31 (Fig. 3). Furthermore, both sisters were carriers of a VUS in *BRIP1* (c.2087C>T p.(Pro696Leu)) (Table 3). Pathogenic variants in *BRIP1* have been associated with increased risk of OC^34–36^, but it remains to be investigated whether the germline variant in *BRIP1* is the cause for the ovarian cancers in both sisters.

Another *ATM* variant identified in our cancer cohort was the pathogenic c.5932G>T variant. This variant was identified in a woman (P-91) diagnosed with BC at age 54. *ATM* c.5932G>T is predicted to be a nonsense variant, p.(Glu1978*), however, this variant has previously been shown to be a splice-affecting variant resulting in skipping of exon 40 and introducing a premature stop codon; p.Ser1974Ilefs*4^37^. This variant has been shown to be associated with HBOC in several studies^16,37,38^.

A third *ATM* variant was the c.8432delA p.(Lys2811Serfs*46). This variant was identified in a patient diagnosed with OC at age 38.

The three variants in *ATM* found in the present study were frameshift variants leading to a premature stop-codon and have been previously identified as disease associated variants identified in the Scandinavian A-T cohort^33^. It has long been debated whether a monoallelic truncating *ATM* variant may increase cancer risks. Some studies indicate that truncating variants lead to increased cancer risk^28,29,31^, whereas others claim that missense variants exerting a dominant negative outcome are responsible for the associated increased cancer risk. In a meta-analysis of *ATM* variants, published by Tavtigian and colleagues (2009)^30^, they found marginal evidence that protein-truncating and splice-junction variants contribute to breast cancer risk, and stronger evidence that some evolutionary rare missense variants increase cancer risk.

The likely pathogenic *CHEK2* c.319+2T>A variant identified in this study has previously been identified in another Norwegian patient diagnosed with thyroid cancer at age 31, BC at 43 and 48. Her family history included both BC and endometrial cancer^39^. Two of the patients in our cohort were carriers of this *CHEK2* variant (P-12 and P-16; Table 2). P-12 was diagnosed with OC at age 27, while P-16 was diagnosed with OC at age 70. Interestingly, P-12 was also heterozygous for a VUS, the novel *CDH1* c.136C>G p.(Leu46Val).

Another interesting *CHEK2* variant is c.470T>C p.(Ile157Thr) in exon 4. This variant is well characterized and proposed as low-penetrant variant which is estimated to give a lifetime BC risk of 18.3%^40^. This variant was identified in P-59, diagnosed with BC at age 58 The variant is however categorized as a VUS, due to the high allele frequency in the Finnish population in gnomAD (2.50%), although this may be in concordance with the low increase in BC risk.

Deleterious variants in *TP53* are the cause of Li-Fraumeni syndrome (LFS), a cancer predisposition syndrome associated with the development of various tumours: soft tissue sarcoma, osteosarcoma, pre-menopausal breast cancer, brain tumours, adrenocortical carcinoma and leukemias^41^. There is also an increased risk for Wilms’ tumour, skin, gastrointestinal, lung, endometrial, ovarian, prostate and gonadal germ cell cancers^41,42^. P-13 was a carrier of the known pathogenic sequence variant c.818G>A p.(Arg273His) in *TP53*^43–46^. However, the patient’s family does not meet the classic LFS criteria nor the revised Chompret criteria for LFS (Fig. 3)^41^. The patient was diagnosed with an early-onset BC (36 years), had a sister diagnosed with Wilms’ tumour at age 6 and a father with a cancer of unknown origin diagnosed at age 55, thereby fulfilling the Birch criteria for LFS-like^47^. Knowledge of her family history was sparse, which might explain why the Li-Fraumeni/Chompret criteria were not met for *TP53* testing. Today, the family would have been offered testing for sequence variants in *TP53*, amongst others. The *TP53* c.818G>A p.(Arg273His) variant, identified in this family, is located at a position in the *TP53* gene which is characterized as a common hotspot for somatic mutations^48^. The variant was identified in 33% of the sequence reads from P-13. Somatic pathogenic sequence variants in *TP53* have been shown to increase in blood of women who have endured chemotherapy treatment^49^. Accordingly, this patient might have a somatic sequence variant. However, a skewed amount of reads may also be due to a technical artefact. Further family studies are needed to determine the nature of this variant.

Although several NGS studies of patients with BC and/or OC have identified LPVs/PVs in the MMR genes^9,12–14,17,23^, we did not identify pathogenic variants in *MLH1, MSH2, MSH6* or *PMS2*. We identified the *MLH1* c.[-28A>G; -7C>T] in three patients. These variants are located in *cis* and have been shown to reduce the expression of MLH1 by 50% from this allele^50^. However, according to gnomAD these variants are identified with a minor allele frequency of 0.8% in the Finnish population. Furthermore, as there is still 50% MLH1 tumour suppressor function from the mutated allele^50^, it may provide a sufficient amount of MLH1 transcripts and accordingly not contribute to an increased cancer risk. Morak *et al*. investigated the promoter region of *MLH1* in 480 patients with colorectal cancer (CRC) and 1150 controls. They identified the variant in an individual with MLH1-proficient CRC and two individuals with non-Lynch syndrome tumours, all part of one of the control groups in the study. Additionally, they found biallelic expression in cDNA from the three individuals with this variant.

*RAD51C* c.1026+5_1026+7delGTA was identified in P-69, diagnosed with OC at age 52 and the family history included BC, OC and prostate cancer. Janatova and colleagues (2015) identified this variant in a patient diagnosed with OC and later endometrial cancer. They classified this variant as likely pathogenic as it affects splicing by causing skipping of exon 8, resulting in a frameshift with an premature stop codon (p.Arg322Serfs*22)^51^. Only one other pathogenic *RAD51C* variant has been identified in the Norwegian population, as far as we know.

A novel *ERCC5* c.67G>T p.(Glu23*) was identified in a woman diagnosed with BC at age 49 (P-44) (Table 2). The variant is predicted to introduce a stop codon, which will lead to transcripts that might be targeted for nonsense mediated mRNA decay (NMD). If ERCC5 is synthesized, it will lack most of the protein sequence. In addition, the variant is predicted to introduce a new cryptic 5′ splice site (ss) one nucleotide up-stream. The outcome of aberrant splicing using this cryptic splice site would lead to skipping of 23 nucleotides and a subsequent frameshift, introducing a premature stop codon (p.(Glu23Tyrfs*2)). Another possibility is that of an alternative translational start site down-stream of this variant, since it is located in the first exon of the gene. However, the next in-frame start-codon is Met169 in exon 5. Usage of this methionine as a start codon has not been reported.

The LPV *FANCM* c.5101C>T, p.(Gln1701*) was identified in two patients; P-8 and P-41 (Table 2). P-8 was diagnosed with BC at 56 years of age and P-41 was diagnosed with BC at age 69. Pathogenic variants in *FANCM*, including this variant, have previously been reported to confer an increased risk of BC^21,52–55^.

One of the patients in the study (P-90) carried the *WRN* variant c.1105C>T p.(Arg369*) and was diagnosed with BC at 57 years of age (Table 2). This variant introduces an early stop codon and has previously been reported in ClinVar and HGMDp as a pathogenic and disease mutation, respectively, in patients with Werner syndrome. Werner syndrome is an autosomal recessive disease characterized by the early appearance of features associated with normal aging and increased cancer risk^56^. Accordingly, heterozygous carriers might have an increased cancer risk^57^. This assumption is supported by another NGS study of breast cancer patients that identified a deleterious *WRN* sequence variant (c.4245dupT, p.(Asp1416*))^13^. In addition, Ding and colleagues (2007) have also reported association between *WRN* and breast cancer^58^.

For some of these variants, such as the variants in *ERCC5* and *WRN*, the link between a heterozygous pathogenic variant and BC/OC is not well defined. For women carrying these variants, there is no clinical benefit from the discovery of these variant as there are currently no management plans or reliable risk data. However, the discovery of such variants in patients with BC/OC may in the future lead to better-documented associations, and subsequently to reliable risk data and management plans for these patients.

NGS gene panels generally has its limitations; variants in non-target regions cannot be detected, some regions have gaps due to insufficient probe coverage, pseudogenes can cause misalignments of reads, repetitive segments can create technical artefacts reported as deletions/insertions, deletions covering entire exons may not be detected, etc. Additional BC and OC cases might have been resolved if we had resequenced the gaps using Sanger sequencing, as well as investigated untranslated regions and regions further out in introns than +/−10 nucleotides. Furthermore, no copy number variation analysis using NGS-data or MLPA was used to investigate these genes; accordingly, large deletions or duplications could go undetected.

The challenge with pseudogenes is well illustrated with the *PMS2* gene, which has several. Amongst these pseudogenes, one in particular confers problems during NGS, the *PMS2CL*. This pseudogene consists of exons almost identical to exon 9 and 11–15, including intronic sequences. Accordingly, the software has difficulties in aligning the sequences to the correct genomic position. Two of our samples, P-56 and P-57 (from the same family), initially seemed to have a deletion of a part of the *PMS2* gene. However, secondary evaluations of reads using IGV revealed that most of the reads aligned with the *PMS2CL* reference sequence. This may be the result of gene conversion between *PMS2* and *PMS2CL*^59^. Gene conversion might mask variants due to faulty alignment of reads to both *PMS2* and *PMS2CL*. Consequently, both genes should therefore be manually investigated in IGV. Alternatively, to prevent overlooking PVs in *PMS2*, examination of *PMS2* cDNA, as proposed by van der Klift and colleagues, could be included in the screening for PVs^60^.

Our current study is starting to reveal the diversity of genetic cancer risk factors in a Norwegian cancer cohort. However, a much larger patient study is warranted to assess the appropriate distribution of variants in Norway. Additionally, several sequence variants were identified, for which the clinical significance is currently unknown. Accordingly, there is a need for robust functional assays to study the biological consequences of these variants. The study demonstrates the necessity for more knowledge from similar studies and the investigation of families with these PVs/LPVs. Increased knowledge may contribute to the development of new and more specific clinical management programs.

## Patients and Methods

### Patients and samples

This study included samples from 101 (P-1–P-101) Norwegian patients from 93 unrelated families (referred to the Department of Medical Genetics at the University Hospital of North Norway) diagnosed with BC and/or OC) (Supplementary Table S1). All patients had previously been screened for PVs in *BRCA1* and *BRCA2*, using Sanger sequencing/NGS and multiplex ligation-dependent probe amplification (MLPA), but no PVs, LPVs or VUSs were identified.

The cancer patients included in this study were divided in three groups, according to how they were recruited. Group 1 (n = 32) and 2 (n = 46) included patients previously tested for PVs in *BRCA1/2* by Sanger sequencing. Samples from these patients were resequenced using the NGS technology. Group 1 represented samples from deceased patients and group 2 samples from surviving patients. Group 3 (n = 23) included patient samples previously sequenced using NGS technology, but where only *BRCA1/2* had been analyzed. The sequence data for the additional 92 genes was available for group 3 patients and were further analyzed in this study.

For group 1 and 2, blood stored in the diagnostic biobank at the department was used. Genomic DNA was extracted using QIAsymphony (QIAGEN, Hilden, Germany) with the QIAsymphony DNA Mini Kit (QIAGEN), according to the manufacturer’s protocol.

### Compliance with Ethical Standards

The project was approved by the Norwegian Regional Ethics Committee (ref. nr. 2016/980) and all experiments were performed in accordance with guidelines/regulations. The committee allowed inclusion of samples from deceased patients (group 1) without informed consent. The committee approved exemption from written informed consent from patients in group 2, where passive informed consent was obtained instead. Written informed consent was obtained from patients in group 3.

### Analysed cancer genes

The TruSight cancer sequencing kit (Illumina, San Diego, CA, USA) containing probes to enrich 94 cancer related genes was used. All 94 genes were scrutinized for nonsense and frameshift variants, and 16 genes previously associated with BC or OC (Table 1) were investigated for all types of sequence variations. We also verified the normal results from the previous screening of *BRCA1/2*.

### Library preparation and sequencing

Patients DNA samples were quantified using the Qubit dsDNA High Sensitivity (HS) assay kit (Invitrogen, Thermo Fisher Scientific, Carlsbad, CA, USA) and measured on a Qubit 3.0 Fluorometer (Invitrogen, Thermo Fisher Scientific) according to manufacturer’s protocol. Quantification of DNA samples was performed prior to DNA tagmentation, before DNA libraries were pooled, and for end-library validation. The HS DNA kit and the 2100 Bioanalyzer (Agilent Technologies, Santa Clara, CA, USA) were used according to manufacturer’s protocol for size-determination of tagmented fragments. Libraries were produced using the TruSight Rapid Capture kit (24 indexes) (Illumina) together with the TruSight Cancer sequencing panel. Sequencing was performed on a MiSeq Sequencer (Illumina).

### Sequencing data analysis

Alignment and variant calling were performed using the MiSeq reporter software (version 2.6.2.3). The MiSeq reporter aligns the sequence reads against the reference genome hg19 using the Burrows-Wheeler Aligner (BWA) and calls variants using the Genome Analysis Toolkit (GATK).

Annotation and filtration of the sequenced variants were done using the Cartagenia Bench NGS software (Agilent). Variants were filtered based on call quality (≥30), genotype quality (≥20), R8 (deletion/insertion after eight mononucleotide or dinucleotide repeats), variant allele frequencies (≥0.2) and read depth (≥18). Variants which passed the quality filters were then filtered based on population allele frequencies (<1% in total populations of ESP6500, ExAC, 1000 genomes Phase 1 and 3, and dbSNP) and position in the gene (exons and up to +/−10 in introns).

The Integrative Genomics Viewer (IGV) (Broad Institute, Cambridge, MA, USA; https://www.broadinstitute.org/igv/) was used for manual inspection of certain regions. These regions included reported gaps (<30 reads) by the analysis software, inspection of variants passed filtering and the entire *PMS2* gene, together with its pseudogene *PMS2CL*. In addition, one position was manually investigated for all samples, chr2:47641560 for variant *MSH2* c.942+3A>T (intron 5). The position of the *MSH2* variant needed manual investigation due to a poly A-stretch, inducing technical deletions/insertions artefacts that might mask this variant^61^.

### Nomenclature

Variants were named following the guidelines proposed by the Human Genome Variation Society (HGVS) nomenclature^62^. Reference sequences used are listed in Table 1, and custom exon numbering was used for *BRCA1* (missing exon 4).

### Classification and Sanger sequencing confirmation

Primers were designed using the Primer 3 software (http://bioinfo.ut.ee/primer3-0.4.0/) and evaluated using SNPCheck3 (www.snpcheck.net/*)*. Primers were excluded if they aligned to sites that covered three or more single nucleotide polymorphism (SNP), if they included SNPs with a minor allele frequency above 0.5% or if SNPs occurred in the last five nucleotides of the primers^63,64^. Primers are listed in Supplementary Table S3. All primers included M13 forward and M13 reverse primer sequences, respectively, for sequencing purposes (M13.F: 5′-tgtaaaacgacggccagt-3′ and M13.R. 5′-caggaaacagctatgacc-3′).

*In silico* evaluation of the variants was done using Alamut® Visual v.2.11.0 (Interactive Biosoftware, Rouen, France), which includes the missense prediction programs Align GVGD, SIFT, MutationTaster and PolyPhen-2. Alamut also contains the splice prediction tools SpliceSiteFinder-like (SSF), MaxEntScan (MES), NNSPLICE, GeneSplicer (GS) and Human Splicing Finder (HSF). In addition, Alamut interactive software provides results and/or links to the following databases used in this study: the Exome Aggregation Consortium (ExAC)/the Genome Aggregation Database (gnomAD), the Exome Variant Server (EVS), the Database of Short Genetic Variation (dbSNP) and ClinVar. The Human Gene Mutation Database Professional (HGMDp) was queried independently.

Classification of variants was performed based on the ACMG guidelines^65^, with some modifications leading to stricter classification criteria.

## Supplementary information

Dataset 1

## Data Availability

The raw sequencing datasets generated during and/or analysed during the current study are not publicly available due to the privacy law/data protection law, which prohibit the disclosure or misuse of information about private individuals. However, screenshots from IGV of the reported sequence variants and surrounding regions can be obtained from the corresponding authors on reasonable request.
