# Supplementary material for: Identifying sequence variants contributing to hereditary breast and ovarian cancer in BRCA1 and BRCA2 negative breast and ovarian cancer patients
Source: Sci Rep. 2019 Dec 27;9:19986. doi: 10.1038/s41598-019-55515-x (PMC6934654; doi:10.1038/s41598-019-55515-x)
Supplement: Supplementary file 1 — Dataset 1 [file 41598_2019_55515_MOESM1_ESM.pdf]

Title: Identifying sequence variants contributing to hereditary breast and ovarian cancer in *BRCA1/2* negative breast and ovarian cancer patients

Authors: Elisabeth Jarhelle*[1,2,3], Hilde Monica Frostad Riise Stensland[1,3], Geir Åsmund Myge Hansen[1,3], Siri Skarsfjord[1], Christoffer Jonsrud[1,3], Monica Ingebrigtsen[1], Nina Strømsvik[1,3,4] and Marijke Van Ghelue*[1,2,3].

Affiliations:
1. Department of Medical Genetics, Division of Child and Adolescent Health, University Hospital of North Norway, Tromsø, Norway.
2. Department of Clinical Medicine, University of Tromsø, Tromsø, Norway.
3. Northern Norway Family Cancer Center, Department of Medical Genetics, University Hospital of North Norway, Tromsø, Norway.
4. Department of Health and Caring Sciences, Western Norway University of Applied Sciences, Bergen, Norway.

Supplementary Table S1. Study cohort. BC = breast cancer; OC = ovarian cancer; CC = cervix cancer. Age at diagnoses for the other diagnoses are listed in parenthesis

Patient	***Diagnosis***	***Other diagnoses***	***Age at diagnosis (other diagnoses)***
P-1	*BC*		*36*
P-2	*BC*		*57*
P-3	*BC*		*63*
P-4	*OC*		*60*
P-5	*OC*		*55*
P-6	*BC*		*65*
P-7	*BC*		*50*
P-8	*BC*		*56*
P-9	*BC*		*44*
P-10	*BC*	*Ventricle cancer*	*55 (64)*
P-11	*OC*		*53*
P-12	*OC*	*Sarcoma (unspecified)*	*27 (61)*
P-13	*BC*		*36*
P-14	*BC*		*35*
P-15	*Bilateral BC*		*74/80*
P-16	*OC*		*70*
P-17	*BC*		*49*
P-18	*OC*	*BC*	*67 (75)*
P-19	*Bilateral BC*		*48/52*
P-20	*BC*		*56*
P-21	*OC*		*52*
P-22	*BC*		*86*
P-23	*BC*		*48*
P-24	*OC*		*57*
P-25	*BC*		*51*
P-26	*Bilateral BC*		*37/47*
P-27	*BC*		*56*
P-28	*OC*		*54*
P-29	*BC*	*Lung cancer*	*39 (58)*
P-30	*OC*		*51*
P-31	*OC*		*47*
P-32	*OC*		*52*
P-33	*BC*	*Colon cancer*	*(47) 57*
P-34	*BC*		*42*
P-35	*Bilateral BC*	*BC*	*41/42 (50)*
P-36	*BC*		*57*
P-37	*BC*		*61*
P-38	*BC*		*48*

Patient	***Diagnosis***	***Other diagnoses***	***Age at diagnosis (other diagnoses)***
P-39	*BC*		*51*
P-40	*BC*		*59*
P-41	*BC*		*69*
P-42	*BC*		*63*
P-43	*BC*		*48*
P-44	*BC*		*49*
P-45	*BC*		*63*
P-46	*BC*		*50*
P-47	*BC*		*50*
P-48	*BC*		*39*
P-49	*BC*		*41*
P-50	*BC*		*51*
P-51	*BC*		*61*
P-52	*OC*		*50*
P-53	*BC*		*38*
P-54	*OC*		*37*
P-55	*OC*		*35*
P-56	*BC*		*61*
P-57	*BC*		*57*
P-58	*Bilateral BC*		*57*
P-59	*BC*		*58*
P-60	*BC*		*61*
P-61	*OC*		*52*
P-62	*OC*	*Skin cancer (unspecified) + CC*	*38 (48 + 65)*
P-63	*BC*		*25*
P-64	*BC*		*35*
P-65	*Bilateral BC*		*51/51*
P-66	*BC*		*53*
P-67	*BC*		*51*
P-68	*OC*	*Bilateral BC*	*55 (58/58)*
P-69	*OC*		*52*
P-70	*BC*		*53*
P-71	*OC*		*48*
P-72	*BC*	*Kidney cancer + Uterus cancer*	*48 (53 + 62)*
P-73	*BC*		*50*
P-74	*BC*		*54*
P-75	*BC*	*Malign melanoma*	*(42) 49*
P-76	*BC*		*55*
P-77	*BC*		*41*
P-78	*BC*		*49*

Patient	*Diagnosis*	*Other diagnoses*	*Age at diagnosis (other diagnoses)*
P-79	*BC*		*55*
P-80	*BC*		*58*
P-81	*BC*		*64*
P-82	*BC*		*37*
P-83	*BC*		*59*
P-84	*BC*		*69*
P-85	*BC*		*68*
P-86	*BC*		*39*
P-87	*BC*		*43*
P-88	*BC*		*48*
P-89	*BC*		*47*
P-90	*BC*		*57*
P-91	*BC*		*54*
P-92	*BC*		*51*
P-93	*BC*		*32*
P-94	*BC*		*55*
P-95	*Bilateral BC*		*57/62*
P-96	*BC*		*59*
P-97	*BC*		*50*
P-98	*BC*		*53*
P-99	*BC*		*52*
P-100	*BC*		*52*
P-101	*BC*		*53*

Supplementary Table S2. Variants that passed filter settings, but classified as benign or likely benign. Variants are named according to Human Genome Variation Society (HGVS) nomenclature. The ClinVar references and the corresponding clinical significance are mainly linked to the condition “Hereditary cancer-predisposing syndrome”, exceptions are marked. O-T: Ocular telangiectasia; BOC: Breast and/or ovarian cancer; LS: Lynch syndrome; PC: prostate cancer; CRC: colorectal, non-polyposis; DM?: disease mutation with some degree of doubt; DFP: Disease-associated polymorphism with additional supporting functional evidence; R: retired from HGMD

Gene	***Variant***	***localization***	***protein change***	***Databases***				***Number of patients***
				dbSNP	***gnomAD***	***ClinVar***	***HGMDp***	
ATM	*c.146C>G*	*Exon 3*	*p.(Ser49Cys)*	*rs1800054*	*ALL: 0.72%; NFE: 1.28%; FIN: 0.26%*	*RCV000128940.4 benign/likely benign*	*CM990201 A-T: DM?*	*2*
ATM	*c.162T>C*	*Exon 3*	*p.(=)*	*rs3218690*	*ALL: 0.18%; NFE: 0.28%; FIN: 0.17%*	*RCV000123737.6 benign/likely benign*	*-*	*1*
ATM	*c.1229T>C*	*Exon 9*	*p.(Val410Ala)*	*rs56128736*	*ALL: 0.21%; NFE: 0.32%; FIN: 0.01%*	*RCV000115136.8 benign/VUS*	*CM035782 O-T: DM?*	*1*
ATM	*c.2119T>C*	*Exon 13*	*p.(Ser707Pro)*	*rs4986761*	*ALL: 0.80%; NFE: 1.20%; FIN: 0.52%*	*RCV000128903.5 benign/likely benign*	*CM013692 BC, ass: DFP*	*2*
ATM	*c.2572T>C*	*Exon 17*	*p.(Phe858Leu)*	*rs1800056*	*ALL: 0.86; NFE: 1.43%; FIN: 0.45%*	*RCV000131019.6 benign/likely benign*	*CM061641 BC, ass: DP*	*4*
ATM	*c.5071A>C*	*Exon 34*	*p.(Ser1691Arg)*	*rs1800059*	*ALL: 0.18%; NFE: 0.29%; FIN: 0.28%*	*RCV000115204.7 benign/likely benign*	*CM980141 A-T: DM?*	*4*
ATM	*c.5793T>C*	*Exon 39*	*p.(=)*	*rs3092910*	*ALL: 0.51%; NFE: 0.65%; FIN: 0.08%*	*RCV000123752.6 likely benign/benign*	*-*	*1*
BRIP1	*c.517C>T*	*Exon 6*	*p.(Arg173Cys)*	*rs4988345*	*ALL: 0.27%; NFE: 0.46%; FIN: 0.19%*	*RCV000129172.5 benign/likely benign*	*CM035889 BC: DM?*	*1*
BRIP1	*c.577G>A*	*Exon 6*	*p.(Val193Ile)*	*rs4988346*	*ALL: 0.36%; NFE: 0.51%; FIN: 0.10%*	*RCV000116161.6 benign*	*-*	*2*
BRIP1	*c.584T>C*	*Exon 6*	*p.(Leu195Pro)*	*rs4988347*	*ALL: 0.19%; NFE: 0.20%; FIN: 0.88%*	*RCV000116162.7 likely benign*	*-*	*1*
BRIP1	*c.890A>G*	*Exon 7*	*p.(Lys297Arg)*	*rs28997570*	*ALL: 0.10%; NFE: 0.17%; FIN: 0.10%*	*RCV000116167.6 benign/likely benign*	*-*	*1*

Gene	*Variant*	*localization*	*protein change*	*Databases*				*Number of patients*
				dbSNP	*gnomAD*	*ClinVar*	*HGMDp*	
BRIP1	*c.2097+7G>A*	*Intron 14*	*p.(?)*	*rs4988352*	*ALL: 0.23%; NFE: 0.41%; FIN: 0.13%*	*RCV000579602.1 likely benign*	*-*	*2*
CDH1	*c.345G>A*	*Exon 3*	*p.(=)*	*rs1801023*	*ALL: 0.36%; NFE: 0.41; FIN: 0.06%*	*RCV000128931.4 benign*	*-*	*1*
CDH1	*c.699C>T*	*Exon 6*	*p.(=)*	*rs115494727*	*ALL: 0.01%; NFE: 0.01%; FIN: 0%*	*RCV000124177.6 likely benign*	*-*	*1*
CDH1	*c.1680G>C*	*Exon 11*	*p.(=)*	*rs35741240*	*ALL: 0.33%; NFE: 0.45%; FIN: 0.20%*	*RCV000132171.5 benign/likely benign*	*-*	*1*
MLH1	*c.[-28A>G; -7C>T]*	*5'UTR*	*p.(?)*	*rs56198082 + rs104894994*	*ALL: 0.17% + 0.13%; NFE: 0.11% + 0.10%; FIN: 0.81% + 0.80%*	*RCV000075059.2 + RCV000075068.2; VUS*[c]	*CR015013 + CR155779; CRC, NP: DM*	*3*
MLH1	*c.790+10A>G*	*Intron 9*	*p.(?)*	*rs182733777*	*ALL: 0.19%; NFE: 0.36%; FIN: 0.13%*	*RCV000075846.4 benign* [b]	*CS086116 CRC: DM?*	*1*
MLH1	*c.803A>G*	*Exon 10*	*p.(Glu268Gly)*	*rs63750650*	*ALL: 0.02%; NFE: 0.02%; FIN: 0.14%*	*RCV000075874.4 benign* [b]	*CM981291 CRC: DM*	*1*
MLH1	*c.1379A>C*	*Exon 12*	*p.(Glu460Ala)*	*rs202038499*	*ALL: 0.01%; NFE: 0.03%; FIN: 0.00%*	*RCV000115459.7 likely benign*	*CM1614470 LS: DM*	*1*
MSH2	*c.23C>T*	*Exon 1*	*p.(Thr8Met)*	*rs17217716*	*ALL: 0.27%; NFE: 0.02%; FIN: 0.14%*	*RCV000076466.6 benign* [b]	*CM001245 CRC: DM?*	*1*
MSH2	*c.339G>A*	*Exon 2*	*p.(=)*	*rs35898375*	*ALL: 0.28%; NFE: 0.46%; FIN: 0.19%*	*RCV000030252.6 benign* [b]	*-*	*2*
MSH2	*c.1680T>C*	*Exon 11*	*p.(=)*	*rs200056411*	*ALL: 0.03%; NFE: 0.07; FIN: 0.02%*	*RCV000076241.5 benign* [b]	*-*	*1*
MSH6	*c.3246G>T*	*Exon 5*	*p.(=)*	*rs3136351*	*ALL: 0.33%; NFE: 0.56%; FIN: 0.39%*	*RCV000126832.6 benign/likely benign*	*-*	*3*
NF1	*c.528T>A*	*Exon 5*	*p.(Asp176Glu)*	*rs112306990*	*ALL: 0.37%; NFE: 0.50%; FIN: 1.17%*	*RCV000129680.2 benign*	*CM077302 NF1: R*	*1*
NF1	*c.1810T>C*	*Exon 16*	*p.(=)*	*rs142712751*	*ALL: 0.13%; NFE: 0.22%; FIN: 0.05%*	*RCV000163286.1 likely benign*	*-*	*1*

Gene	***Variant***	***localization***	***protein change***	***Databases***				***Number of patients***
				dbSNP	***gnomAD***	***ClinVar***	***HGMDp***	
PALB2	*c.1194G>A*	*Exon 4*	*p.(=)*	*rs61755173*	*ALL: 0.08%; NFE: 0.13%; FIN: 0.00%*	*RCV000114462.9 benign/likely benign*	*-*	*1*
PALB2	*c.1572A>G*	*Exon 4*	*p.(=)*	*rs45472400*	*ALL: 0.32%; NFE: 0.49%; FIN: 0.17%*	*RCV000114480.9 benign/likely benign*	*-*	*3*
PALB2	*c.2794G>A*	*Exon 8*	*p.(Val932Met)*	*rs45624036*	*ALL: 0.52%; NFE: 0.64%; FIN: 1.66%*	*RCV000114559.11 benign/likely benign*	*CM112100 BC: DM?*	*3*
PALB2	*c.2816T>G*	*Exon 8*	*p.(Leu939Trp)*	*rs45478192*	*ALL: 0.10%; NFE: 0.17%; FIN: 0.00%*	*RCV000114561.9 likely benign/VUS*	*CM105609 BC: DM?*	*1*
PMS2	*c.52A>G*	*Exon 2*	*p.(Ile18Val)*	*rs63750123*	*ALL: 0.91%; NFE: 1.13%; FIN: 3.15%*	*RCV000144650.2 likely benign* [b]	*CM143109 PC: DM?*	*3*
PMS2	*c.1437C>G*	*Exon 11*	*p.(His479Gln)*	*rs63750685*	*ALL: 0.46%; NFE: 0.39%; FIN: 1.12%*	*RCV000162366.2 benign*	*CM1612932 BOC: DM?*	*2*
PMS2	*c.1569C>G*	*Exon 11*	*p.(=)*	*rs141458772*	*ALL: 0.62%; NFE: 0.42%; FIN: 4.13%*	*RCV000076816.7 likely benign* [b]	*-*	*3*
PMS2	*c.1688G>T*	*Exon 11*	*p.(Arg563Leu)*	*rs63750668*	*ALL: 0.64%; NFE: 0.91%; FIN: 0.70%*	*RCV000076820.7 likely benign* [b]	*CM061908 CRC: DM?*	*2*
RAD51C	*c.376G>A*	*Exon 2*	*p.(Ala126Thr)*	*rs61758784*	*ALL: 0.35%; NFE: 0.54%; FIN: 0.03%*	*RCV000411970.1 likely benign* [a]	*-*	*3*
RAD51C	*c.790G>A*	*Exon 5*	*p.(Gly264Ser)*	*rs147241704*	*ALL: 0.17%; NFE: 0.34%; FIN: 0.06%*	*RCV000116180.6; VUS*	*CM123214; OC: DFP*	*1*
RAD51C	*c.859A>G*	*Exon 6*	*p.(Thr287Ala)*	*rs28363317*	*ALL: 0.57%; NFE: 0.96%; FIN: 0.05%*	*RCV000411553.1 likely benign* [a]	*CM1010198 BOC: DM?*	*1*

[a] Breast-ovarian cancer, familial 3

[b] Lynch syndrome reviewed by expert panel (InSiGHT)

Supplementary Table S3. PCR primers used for Sanger verification of pathogenic/likely pathogenic variants and a selection of variants of unknown clinical significance (VUS). Ex= exon. Both M13 forward and M13 reverse primer sequences were added to the 5' end of the respective primers: forward 5'-tgtaaaacgacggccagt-3' and reverse 5'-caggaaacagctatgacc-3'.

Gene + region	***Forward primer 5' →3'***	***Reverse primer 5' →3'***	***Product size (bp)***
ATM ex21-22	*CAGGCATCTAACAAAGGAGAGG*	*GGCACACCGTATATACTCAACA*	*450*
ATM ex40	*TTTTGTTTGCCACCTTCATTAG*	*TCAGTTTAAAAATCACATGGCATC*	*298*
ATM ex58	*TTCCCTGTCCAGACTGTTAGC*	*TGCCAAACAACAAAGTGCTC*	*363*
BRIP1 ex14	*TTGTTAAACTTGTTGCTTGATCTTTT*	*CTTGTTGCCTCTACCCTAGGAA*	*379*
CHEK2 ex2	*AAACTCCAGCCAGTCCTCTC*	*GCTCCCATCCTGTGACATGT*	*389*
CHEK2 ex4	*GGAGAGCTGGTAATTTGGTCA*	*TCCTCCTATGAGAGAGTGGAAAA*	*298*
ERCC5 ex1	*TGCAGTCCGTCGTAGAAGAA*	*TCATCCTGCAGATGCCACT*	*250*
FANCF ex1	*CTAACTGCCCTGGAGACCTG*	*TTTGGACACACGAAGGCATA*	*243*
FANCM ex20	*GCAAAGGCCAATCAAGTGAA*	*AAGGGTGGTGTGGTAGACTG*	*448*
MLH1 ex1	*TGACTGGCATTCAAGCTGTC*	*ATGCGCTGTACATGCCTCT*	*389*
NBN ex5	*TGTCAGATAGTCACTCCGTTTACAA*	*TCCCAAAATGAAATACGTTAACAA*	*297*
RAD51C ex8	*AATGAGTTTGGTCATCTGAACTTTT*	*CAAATATGCTTGCTGCCTACAG*	*247*
TP53 ex8	*TTGGGAGTAGATGGAGCCTG*	*GGTGATAAAAGTGAATCTGAGGC*	*300*
WRN ex9	*GAACTGAGGCCCAGCAATAA*	*CAAAATTTGGAGTTCATGTTCTGT*	*294*
